# Good performances but short lasting efficacy of Actellic 50 EC Indoor Residual Spraying (IRS) on malaria transmission in Benin, West Africa

**DOI:** 10.1186/1756-3305-7-256

**Published:** 2014-05-30

**Authors:** Rock Aïkpon, Michel Sèzonlin, Filémon Tokponon, Mariam Okè, Olivier Oussou, Frédéric Oké-Agbo, Raymond Beach, Martin Akogbéto

**Affiliations:** 1 Centre de Recherche Entomologique de Cotonou (CREC), 06 BP 2604 Cotonou, Bénin; 2 Faculté des Sciences et Techniques, Université d’Abomey Calavi, Abomey Calavi, Bénin; 3 National Malaria Control Programm (NMCP), Ministry of Health, Cotonou, Benin; 4 Center of Desease Control, Atlanta, USA

**Keywords:** IRS, Pirimiphos methyl, Efficacy, Malaria, Benin

## Abstract

**Background:**

The National Malaria Control Program (NMCP) has been using pirimiphos methyl for the first time for indoor residual spraying (IRS) in Benin. The first round was a success with a significant decrease of entomological indicators of malaria transmission in the treated districts. We present the results of the entomological impact on malaria transmission. Entomologic parameters in the control area were compared with those in intervention sites.

**Methods:**

Mosquito collections were carried out in three districts in the Atacora-Dongo region of which two were treated with pirimiphos methyl (Actellic 50EC) (Tanguiéta and Kouandé) and the untreated (Copargo) served as control. *Anopheles gambiae* s.l. populations were sampled monthly by human landing catch. In addition, window exit traps and pyrethrum spray catches were performed to assess exophagic behavior of Anopheles vectors. In the three districts, mosquito collections were organized to follow the impact of pirimiphos methyl IRS on malaria transmission and possible changes in the behavior of mosquitoes. The residual activity of pirimiphos methyl in the treated walls was also assessed using WHO bioassay test.

**Results:**

A significant reduction (94.25%) in human biting rate was recorded in treated districts where an inhabitant received less than 1 bite of *An. gambiae* per night. During this same time, the entomological inoculation rate (EIR) dramatically declined in the treated area (99.24% reduction). We also noted a significant reduction in longevity of the vectors and an increase in exophily induced by pirimiphos methyl on *An. gambiae*. However, no significant impact was found on the blood feeding rate. Otherwise, the low residual activity of Actellic 50 EC, which is three months, is a disadvantage.

**Conclusion:**

Pirimiphos methyl was found to be effective for IRS in Benin. However, because of the low persistence of Actellic 50EC used in this study on the treated walls, the recourse to another more residual formulation of pirimiphos methyl is required.

## Background

Malaria is a major public health problem and *Anopheles gambiae* is one of the major vectors of this disease in sub-Saharan Africa [1]. The current effective vector control tools include the use of Long Lasting Insecticide Nets (LLIN) and Indoor Residual Spraying (IRS) [2]. In sub-Saharan Africa and southern Asia, these two methods have shown good results [3,4] but they have their drawbacks.

The main problem with ITNs and IRS is the development of insecticide resistance, particularly pyrethroid-resistance, which has been demonstrated in several populations of *Anopheles gambiae*[5-8]. In the past decade, the emergence of resistance in populations of *An. gambiae* to common classes of insecticides used in public health has been reported in many countries in Africa, including Côte d’Ivoire [5], Kenya [9], Benin [10,11], Niger [12], Burkina Faso [13], Mali [14], Nigeria [15], South Africa [16] and Cameroon [17]. In recent reports [18] widespread distribution of pyrethroid resistance in *An. gambiae* was shown in southern Benin, and there was a significant increased level of the *kdr* mutation, which remains the major resistance mechanism detected. The lowest frequency of *Ace-1*^*R*^ was recorded during the same study and may be a sign of encouragement to use carbamates or organophosphates as alternative insecticides to pyrethroids for IRS in Benin. However, Aïkpon and others [19] have recently demonstrated a decrease of *An. gambiae* susceptibility to bendiocarb after the IRS implementation using bendiocarb in Atacora region in Benin.

In another study [20], which included six months evaluation of various insecticides in experimental huts, three insecticides (Sumithion 40 WP [Fenitrothion]; Actellic EC [Pirimiphos methyl]; and Ficam M [bendiocarb, 800 g/kg]) were effective against *Anopheles*. However, bendiocarb and pirimiphos methyl are the two products that the National Malaria Control Program (NMCP) has selected for the IRS campaign in 2013 in Atacora region in Benin. This study was conducted at the experimental hut level and it is difficult to extrapolate from these results what might happen at a larger-scale community level [20,21].

The present study aims to evaluate the persistence of the biological efficacy of Actelic 50 EC IRS and it entomologic impact on malaria transmission after large-scale implementation in areas of high resistance to pyrethroids in *An. gambiae*.

## Methods

### Study area

**Figure 1:**
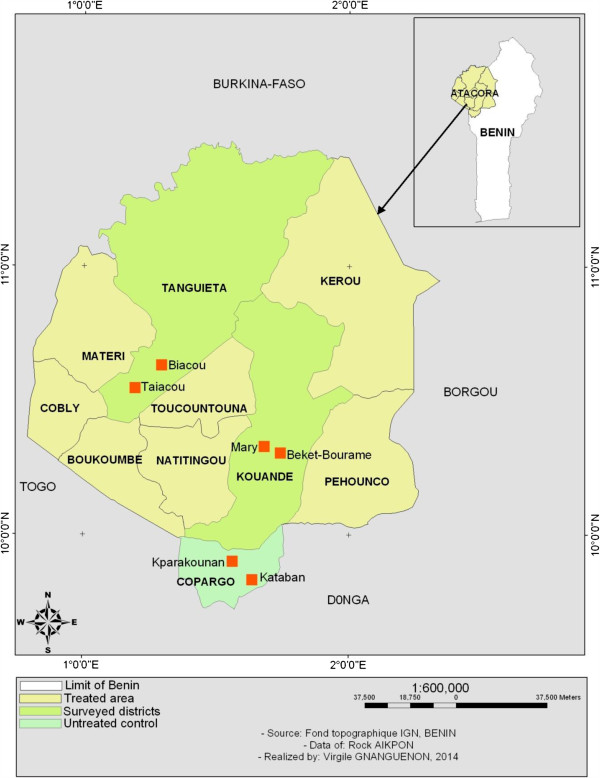
Map of the study area.

The study was carried out in Atacora-Donga region located in the North-west of Benin and includes three districts: Kouandé, Tanguiéta and Copargo (Figure 1). The three districts covered 7,543 km2 and had an estimated population of 287,935 in 2012. Atacora-Donga region has a sub-equatorial type climate with one dry season (December-May) and only one rainy season (June to November). The annual mean rainfall is 1,300 mm and the mean monthly temperature ranges between 22 and 33°C. The region is irrigated by three major rivers: the Mekrou, the Pendjari and the Alibori. The major economic activity is agriculture and it is characterized by the production of cotton and millet where various classes of pesticides are used for pest control.

### Indoor residual spraying (IRS) campaigns 2013

Pirimiphos methyl was selected for spraying on the walls for 2013 IRS campaign in Kouandé, Tanguiéta districts. Studies have shown that this insecticide was efficient in phase II evaluations against malaria vectors [20]. ACTELLIC 50 EC (Syngenta) was therefore applied. The recommended dilution rate for residual application for the control of mosquitoes is 1-2 g of active ingredient per square meter of spray surface. Thus, 50-100 ml of ACTELLIC 50 EC is needed in 1 liter of spray. The 15-liter-capacity H. D. Hudson Manufacturing Company (Chicago, IL) 67422 AD, Hudson X-pert spray pumps, recommended by the WHO for use in IRS, was used. One liter (1000 ml) of ACTELLIC 50EC was mixed in 10 liters of clean water in the spray can and pressurized to 55 Psi. The application rate was 1 m in 2.2 or 4.5 s for 2 m height of wall [22]. Only one round of IRS was carried out per year in the beginning of the rainy season in June. The IRS operation was performed by volunteers chosen from the local community who were trained by the PMI IRS partner (Abt associate team). According to Abt associate, the coverage rate was more than 90% of the households in the target districts. Copargo district, which has similar characteristics to the treated districts (climate, agricultural practices, LLIN coverage) was selected as control and was not treated. Twenty km separate the control (untreated) villages from the treated districts.

### Mosquito sampling

#### Human landing catch

In each district, two villages were selected, and two houses were chosen per village for mosquito collection to monitor malaria transmission. Monthly, mosquito collections were carried out from 6 p.m. to 6 a.m. inside and outside houses using a mouth aspirator, by human volunteers who had previously given consent. Two nights of mosquito collections a month were carried out for three months. A total of six night catches were conducted in each district. These catches were made from June to August 2013 (after IRS intervention) during the recommended duration of effective action of pirimiphos methyl by WHOPES, which is between 2 and 3 months. In each district, two houses and four collectors were selected per area for the collection of mosquitoes. The recorded data were used to assess the aggressiveness (HBR), the physiological age and the entomological inoculation rate vectors (EIR).

#### Exit window trap catch

Moreover, in order to assess the impact of interventions on exit induced by the presence of Actellic 50 EC, we sampled mosquitoes using window exit traps and morning pyrethrum spray catches (PSC). Therefore, in each area, eight bedrooms were selected for mosquito collection in the morning. Exit traps were put over the windows of every bedroom retained. Mosquitoes were collected for 2 nights each month from 6 p.m. to 6 a.m. next day. The houses where the traps were set were selected based on the number of people who slept in them (01 sleeper per hut). The houses were built of mud and wood with a sheet-metal roof and the eve was tight. The area between the upper walls and the roof is closed.

The exit window traps used were made of a terylene netting mounted on a 30 cm cubical metallic frame. The entrance side was drawn into a truncated cone with a 2.5 cm diameter hole at its apex, which was 2.5 cm from the opposite face of the trap. In addition to that, mosquitoes resting in the house were collected by Morning Spray Catch (MSC) from 7 a.m. to 9 a.m. The collections from the window traps were done in the morning using a mouth aspirator. Morning pyrethrum spray catches were done using pyrethrum spray and white canvas spread on the floor to collect knocked down mosquitoes. These two sampling methods led to an accurate estimation of the total density of mosquito species in the treated houses and the proportion of female mosquitoes exiting from the houses. Exit rate is estimated by mosquitoes that have escaped treated walls and have been retained in the exit window traps. *Anopheles* mosquitoes collected were classified according to the state of their abdomens.

#### Bioassay Test

The residual activity of ACTELLIC 50 EC on the sprayed surface was monitored for a period of 4 months using WHO cone tests [23].

#### Biological materials

The susceptible strain “Kisumu” of *An. gambiae* originate from Kenya and were bred and maintained in the insectary of the Entomological Research Centre of Cotonou (CREC) and were used for the bioassays. A local population of *An. gambiae* from Atacora, collected as larvae and raised to adulthood in the insectary were also used.

#### Cone bioassay procedure

Bioassays were carried out monthly on walls of twelve houses randomly selected in the study area. Untreated surfaces were used as control. The efficacy and the residual life of the Actellic 50 EC on the treated wall (mud and cement) was evaluated using WHO cone tests [23]. This test consists of introducing ten to fifteen unfed two to five-day-old female mosquitoes into a plexiglas cone attached to the insecticide-treated wall for 30 minutes. After exposure, the mosquitoes were placed in small cups, provided with sugar solution and maintained at 27 ± 2°C with a relative humidity of 80 ± 10% for 24 hours to assess delayed mortality. Tests were considered as invalid and repeated when control mortalities exceeded 20%. When control mortalities were less than 20%, but exceeded 5%, a correction of mortality was made using Abbot’s formula [24].

### Laboratory processing

After each night catch, Anophelines were morphologically identified to species using taxonomic keys of Gillies & De Meillon [25] and Gillies & Coetzie [26]. Ovaries from randomly selected female *An.gambiae s.l*. specimens captured on human landing catches were dissected to determine parity rate, by observing the degree of coiling of ovarian tracheoles [27]. Mosquito infectivity rates were determined from head and thorax of all female anopheline specimens by enzyme-linked immunosorbent assay (ELISA) using monoclonal antibodies against *Plasmodium falciparum* circumsporozoite protein (CSP) as described by Wirtz *et al*. [28]. The carcass of these females (abdomens, wing and legs) were stored in individual tubes with silicagel and preserved at −20°C in the laboratory for identification of species and characterization of molecular forms within the *An.gambiae* complex as previously described [29,30].

### Data analysis

The human biting rate [number of bites/man/night] (ma), the sporozoïte rate (Is) and the entomological inoculation rate (EIR) were determined. The percentages of reduction of biting rate (HBR) and EIR in the treated districts compared to the control were evaluated. Parturity rates, exophily and blood feeding rate were calculated. Comparisons of these rates were made by the Chi-square test.

### Ethical consideration

Ethical approval for this study was granted by the Ethical Committee of the Ministry of Health in Benin. The mosquito collectors gave prior informed consent and they were vaccinated against yellow fever. They were also subjected to regular medical check-ups with preventive treatments of malaria.

## Results

### Bioassay tests

**Figure 2:**
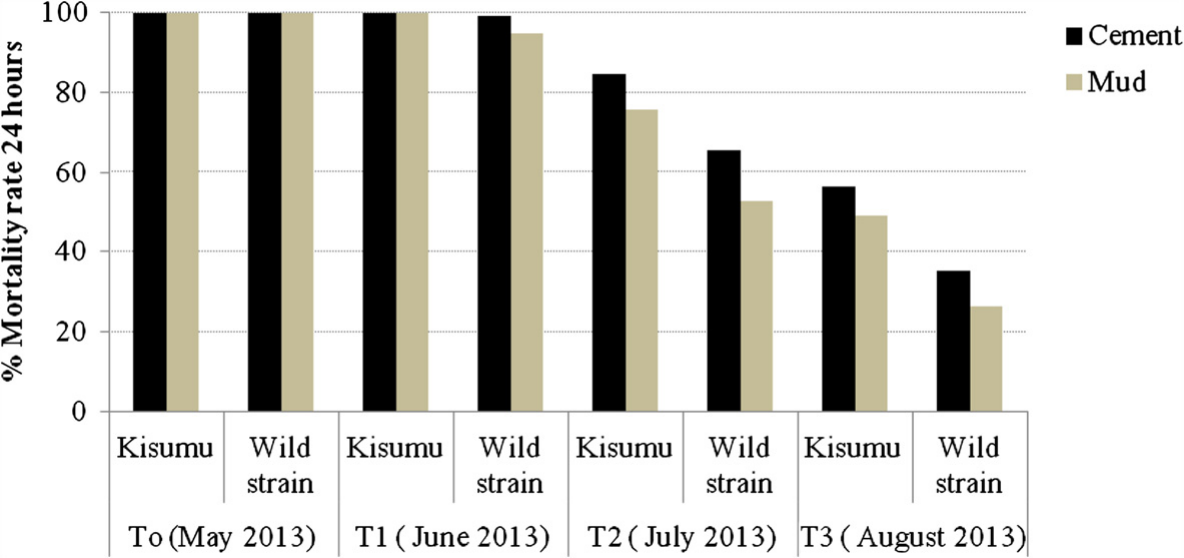
**Three months residual efficacy of Actellic 50 EC indoor residual spraying in Atacora.** (T_0_ = 24 hours after treatement; T_1_ = 30 days after treatement; T_2_ = 60 days after treatement; T_3_ = 90 days after treatement).

The results of bioassay tests on cement and mud surfaces are shown in Figure 2. A total of 1440 wild strain female *An. gambaie s.l* and 1512 *An. gambiae s.s*. susceptible strain Kisumu were exposed on the treated surface during the 4 month period. The bioassay test was conducted monthly from May to August 2013. From T_0_ (24 hours after spray operation), the percentage mortalities after 24 h of observation were 100%. However, after one month, the percentage mortality for wild strain *An. gambiae* was 99% on cement and 94% on mud before a gradual decline (Figure 2). The residual effect of Actellic 50 EC (>80% mortality in cone bioassays) lasted three months. Comparing the persistence of Actellic 50 EC on the cement and mud wall surfaces showed a significant difference (P< 0.05). At 3 months, the mortality of Kisumu has decreased to 56.5% on cement wall surfaces against 49% on mud wall surfaces.

#### *Anopheles gambiae s. l* species identification

**Figure 3:**
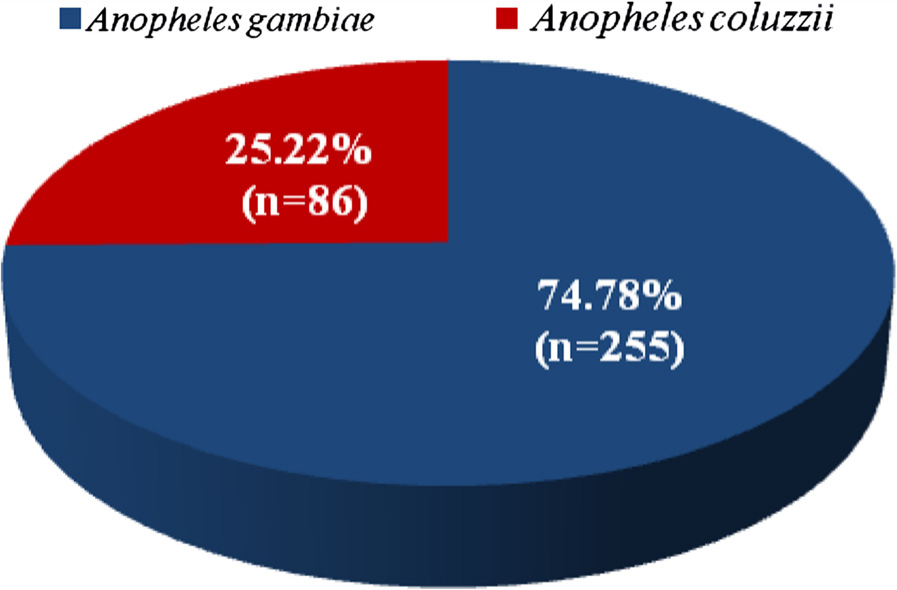
***An. gambiae *** **complex composition in the study area.**

A total of 341 *An. gambiae s.l* mosquitoes were analyzed for species identification. *An. gambiae s.s*. and *An. coluzzii* are sympatric in the study area. However, *An. gambiae* was predominant (74.78%; n = 255) (Figure 3).

#### Impact of IRS on HBR of *An. gambiae*

The results of our research show a lower human biting rate (HBR) in the treated area. During the study period, the *An. gambiae* HBR was 0.55 bite/person/night in intervention areas against 9.56 bites/person/night in the control. The reduction of human biting rate was 94.25% (Table 1).

**Table 1:** Entomological Inoculation Rate (EIR) and Human Biting Rate (HBR) observed in intervention area and the control

**Variables**	**Treated districts**	**Control**	**% reduction**
	**(Tanguiéta, Kouandé)**	**(Copargo)**	
Total	35	306	-
Person night	64	32	-
HBR	0,55	9,56	94,25
S%	0,03	0,22	-
EIR (b/m/n)	0,016	2,11	99,24
EIR/month	0,48	63,3	-

S% = Sporozoite index.

HBR = Human Bite Rate.

EIR (b/m/n) = Entomological Inoculating Rate (infected bite/man/night).

#### Impact of IRS on EIR of *An. gambiae*

The EIR in the control area was 2.11 infected bites/person/night against 0.016 infected bites/person/night in the treated districts (Table 1). In the control area, the rate was very high (63.3 infected bites/man/month). The comparison of EIR observed in treated areas and the control revealed a dramatic reduction of 99.24% in treated districts.

### Impact of IRS on longevity of *An. gambiae*

**Figure 4:**
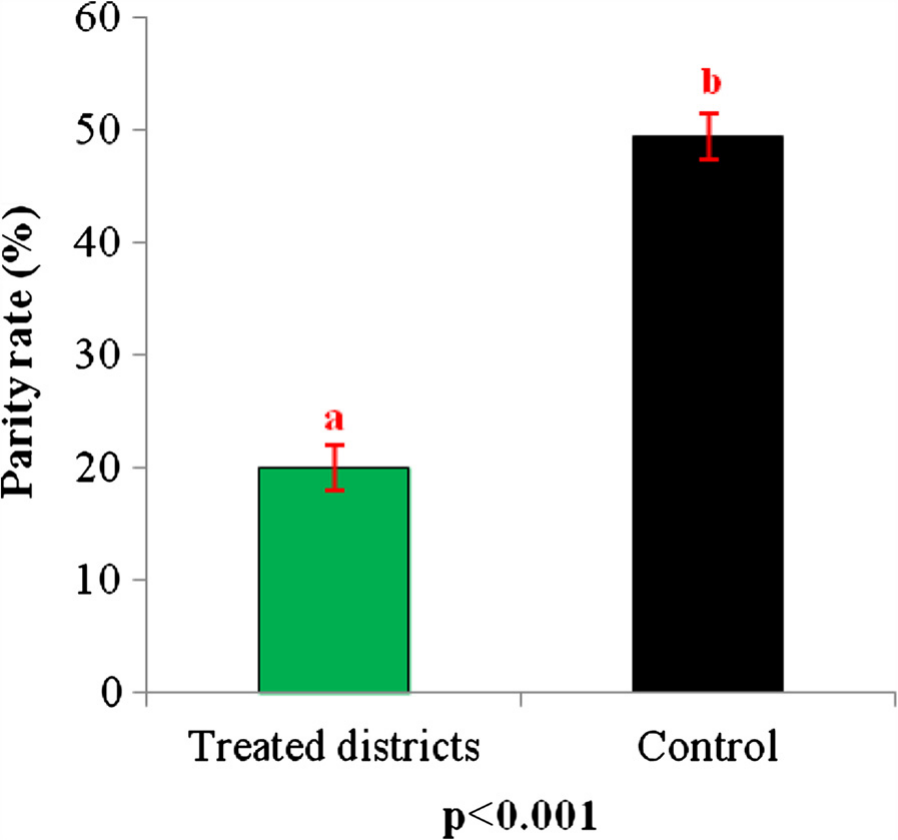
**Parity rate of *Anopheles gambiae s.l. *** **collected in treated area and in control.**

A total of 131 ovaries of *An. gambiae* were dissected. The parity rate of *An. gambiae* was 20% in the treated districts against 49.41% in the control (Figure 4). We observed a significant reduction of longevity (p<0.001). The IRS area was not conducive for mosquito survival.

### Impact of IRS on *An. gambiae* blood feeding rate in treated districts and the control

**Figure 5:**
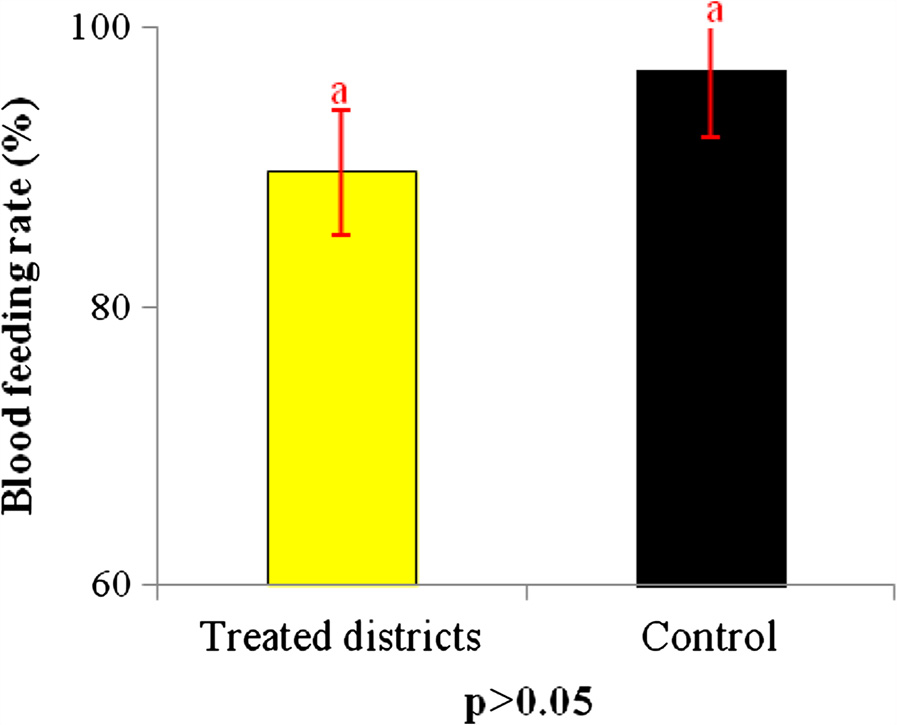
**Percentage of blood feeding *An. gambiae *** **collected in IRS by Pyrethrum Spray Catch (PSC) and in exit windows traps in intervention area and the control.**

The blood feeding rate observed in the treated areas was 89.47% (17/19) against 96.76% (118/122) in the control (Figure 5). The difference was not significant. Despite IRS implementation, a non-negligible proportion of mosquitoes fed on humans.

### Impact of IRS in induced exophily in *An. gambiae* in localities under IRS and the control

The exit rate of *An. gambiae* in the control was 14%. Compared to the control, there is a significant increase of exit rate of *An. gambiae* in the treated districts. In those districts, the exit rate was 100%. This shows that the mosquitoes that enter houses do not manage to stay there and are obliged to exit houses.

## Discussion

The strategy of indoor residual spraying (IRS) with pirimiphos methyl implemented by the National Malaria Control Program (NMCP) in the Atacora region had a great impact on malaria transmission. In the districts under intervention, the density of *An. gambiae* (human biting rate) and the entomological inoculation rate (EIR) were significantly reduced.

In this study, two members of *An. gambiae* complex were found in sympatry (*An. gambiae* and *An. coluzzii*) and their distribution agree with previous findings in Benin that reported both M and S forms with the predominance of S forms in savannah areas [31].

The results have shown that the biting rate of *Anopheles* dropped drastically in treated districts compared to the control. This drastic drop is due to the lethal effect of pirimiphos methyl on the anophelines resistant to pyrethroids [20].

Indeed, comparing the number of *An. gambiae* bites that a person receives in one night in the treated districts and in the control, the rates were significantly reduced. These results were consistent with those of Akogbéto *et al*. [32] and Ossè *et al*. [33] with bendiocarb IRS in the south of Benin.

**Figure 6:**
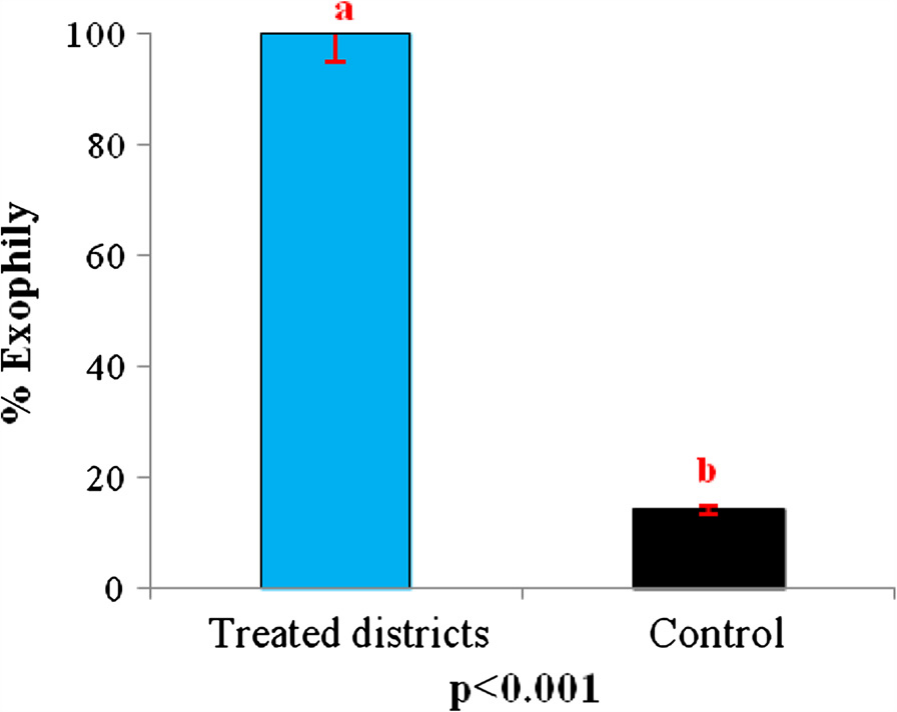
**Exit rate for *Anopheles gambiae *** **from pirimiphos methyl treated walls.**

The unpleasant atmosphere created by the presence of pirimiphos methyl on the walls inside houses is harmful to the mosquitoes. This atmosphere results in an increase in the exit rate in the treated districts (Figure 6). Thus, some *Anopheles* mosquitoes that managed to enter houses failed to obtain blood meals before exiting. However, some succeeded in obtaining blood meals inside houses (Figure 5). The proportion of fed *An. gambiae* collectedin window exit traps in treated districts was not significantly different from that observed in the untreated area (control). When mosquitoes enter the houses, even those houses that are treated, they go directly to their host to obtain a blood meal before resting on walls or seeking to escape if the houses are treated. This situation was found in experimental huts for many insecticides [20,21]. This finding explains why we have proposed that the NMCP always invite communities who are protected by IRS to add sleeping under mosquito nets to supplement malaria control efforts.

The EIR was low in treated districts. This shows that the IRS performed in those districts were a great success. Indeed, we had a reduction of 99.24% of EIR. Despite the effectiveness of IRS, this method has its limitations. A major concern is the low residual activity of actellic 50 EC, which is a maximum of three months. This confirms the the effective minimum duration action of 2–3 months recommended by WHOPES. The period of transmission of malaria is six months in Atacora (from June to November). The low persistence of Actellic 50 EC cannot cover all the periods of transmission. For that reason, we have to suggest to the NMCP, the use of the CS formulation (Actellic CS) for the future IRS campaigns in Atacora. Indeed this formulation recently received the ratification of the WHOPES and can be used in Benin.

## Conclusion

The use of pirimiphos methyl in IRS in Atacora department with the PMI support had a significant impact on malaria transmission in the treated areas. The results show that in intervention areas, the human biting rate of mosquitoes dropped spectacularly. A reduction of 99.24% in the entomological inoculation rate was recorded, due to the decline in the lifespan of *An. gambiae* and high exophily induced by pirimiphos methyl on mosquitoes. However, the limitation that consists of the low residual activity of Actellic 50 EC remains to be resolved, which can be achieved by a change of the formulation.

## Competing interests

The authors declare that they have no competing interests.

## Authors’ contributions

RA, MS, FT, MO, OO, FOA, RM and MA designed the study. RA, FT, OO, MO and MA carried out the field activities. RA drafted the manuscript and analyzed the data. MA and FOA critically revised the manuscript. MA conceived and designed the study and revised the manuscript for intellectual content. All authors read and approved the final manuscript.
